# Molecular pathology testing for non-small cell lung cancer: an observational study of elements currently present in request forms and result reports and the opinion of different stakeholders

**DOI:** 10.1186/s12885-022-09798-5

**Published:** 2022-07-06

**Authors:** Kelly Dufraing, Kaat Van Casteren, Joke Breyne, Nicky D’Haene, Claude Van Campenhout, Sara Vander Borght, Karen Zwaenepoel, Etienne Rouleau, Ed Schuuring, Jan von der Thüsen, Elisabeth Dequeker

**Affiliations:** 1 Department of Public Health and Primary Care, University of Leuven, Biomedical Quality Assurance Research Unit, Leuven, Belgium; 2 Laboratory of Pathological Anatomy, Antwerp University Hospital, Edegem, Belgium; 3 Center for Oncological Research Antwerp (CORE), University of Antwerp, Wilrijk, Belgium; 4 Department of Molecular Diagnostics, AZ Delta Roeselare Menen, Roeselare, Belgium; 5 Department of Pathology, Erasme University Hospital, Brussels, Belgium; 6 Medical Biology and Pathology, Gustave Roussy, Paris, France; 7 Department of Pathology, University of Groningen, University Medical Center Groningen (UMCG), Groningen, the Netherlands; 8 Department of Pathology, Erasmus Medical Center, Rotterdam, the Netherlands; 9 Department of Public Health and Primary Care, University of Leuven, Biomedical Quality Assurance Research Unit, Leuven, Belgium

**Keywords:** Non-small-cell lung cancer, Molecular pathology, Test requesting, Test report, Pre-analytical phase, Post-analytical phase

## Abstract

**Background:**

For patients with non-small cell lung cancer (NSCLC), targeted therapies are becoming part of the standard treatment. It is of question which information the clinicians provide on test requests and how the laboratories adapt test conclusions to this knowledge and regulations.

**Methods:**

This study consisted of two components; 1) checking the presence of pre-defined elements (administrative and key for therapy-choice) on completed requests and corresponding reports in Belgian laboratories, both for tissue- and liquid biopsy (LB)-testing and b) opinion analysis from Belgian pathologists/molecular biologists and clinicians during national pathology/oncology meetings.

**Results:**

Data from 4 out of 6 Belgian laboratories with ISO-accreditation for LB-testing were analyzed, of which 75% were university hospitals. On the scored requests (*N* = 4), 12 out of 19 ISO-required elements were present for tissue and 11 for LB-testing. Especially relevant patient history, such as line of therapy (for LB), tumor histology and the reason for testing were lacking. Similarly, 11 and 9 out of 18 elements were present in the reports (*N* = 4) for tissue and LB, respectively.

Elements that pathologists/molecular biologists (*N* = 18) were missing on the request were the initial activating mutation, previous therapies, a clinical question and testing-related information. For reporting, an item considered important by both groups is the clinical interpretation of the test result. In addition, clinicians (*N* = 28) indicated that they also wish to read the percentage of neoplastic cells.

**Conclusions:**

Communication flows between the laboratory and the clinician, together with possible pitfalls were identified. Based on the study results, templates for complete requesting and reporting were proposed.

**Supplementary Information:**

The online version contains supplementary material available at 10.1186/s12885-022-09798-5.

## Background

For patients with metastatic non-small cell lung cancer (NSCLC), targeted therapies have taken over the position as the standard treatment for tumors with susceptible genomic alterations. For example, *EGFR* tyrosine kinase inhibitors (EGFR-TKIs) are now the first-line treatment for patients with NSCLC with a confirmed actionable *EGFR* variant. These shifts in treatment options require a concomitant expansion of knowledge for clinicians. It is of interest which information regarding the lines of treatment the clinicians provide on their test request forms and how the laboratories correspondingly adapt their reports, including the test conclusion, to this information. Effective communication between the molecular pathology laboratory and the test ordering clinician is thus indispensable for correct test interpretation, and by consequence perhaps also for administering the correct therapy [1].

The laboratory can only perform the correct test and correctly interpret the result when the right question is provided together with the necessary background information. Therefore, laboratories could create a request form template that stimulates clinicians to submit the correct and complete information. Although this request form template might contain the minimally required elements to be filled in, it is not known whether the clinician truly completes all fields and whether they are appropriately interpreted. Studies in the field of hereditary cancer have indeed indicated problems with form completeness [2, 3]. In addition to the completeness of test requests, adequate reporting of the test result by the laboratory is in turn important for efficient communication to the clinician. Although guidelines and checklists on the elements of the test report have been published, these are rather general and do not always fully apply to NSCLC nor are they applicable to multiple testing methods [4]. Furthermore, it remains unclear whether clinicians find each element on the report useful and whether they correctly translate the report to the best treatment decision [5]. For example, a study by Heller et al. indicated that 39% (*N* = 91) of the clinicians did not fully understand the pathology testing results in vulvovaginal disease [6]. Another study by Lubin et al. in the field of hereditary diseases confirms these opinions from clinicians [7].

NSCLC is a complex disease which is subject to significant and progressive change in the tumor genome. Despite initial responses, most patients show disease progression within one to two years after treatment with first-, second- and even third- generation EGFR-TKIs. The most common mechanism of resistance is the development of an additional *EGFR*-T790M variant in exon 20. Phase II and III trials with osimertinib (Tagrisso©, AstraZeneca), a third-generation EGFR-TKI, demonstrated an objective response rate (ORR) of 60–70% and median progression-free survival (mPFS) of 10–11 months in *EGFR*-T790M-positive tumors. Based on the results of the phase III FLAURA trial, the use of osimertinib for the first-line treatment of patients with inoperable or recurrent *EGFR* mutation-positive NSCLC was approved in 2018 [8]. It is important that clinicians are aware of such changes in sequencing of preferential treatment, since this might impact the process of requesting and/or reporting flow of biomarker testing.

Several flow charts of biomarker testing for NSCLC are used in practice. For example, a laboratory can opt for simultaneous testing of the different markers (*EGFR*, ALK, ROS1 and PD-L1) or for sequential testing. For the latter, the test results for the various biomarkers become available at different times. This might also impact reporting of results; thus, do laboratories have one integrated report with one conclusion regarding therapy for all biomarkers, or a separate report for each marker? If the latter is the case, it is debatable whether clinicians are aware that they need to wait for all information before starting the treatment.

Tissue biopsies are currently standard practice in the management of cancer patients. However, sometimes retrieving these tissue samples carries considerable risk to the patient. Therefore, the use of liquid biopsies (blood, urine, saliva, …) as a complement to tissue biopsies is of great interest, especially for repeated *EGFR*-T790M testing [9, 10]. Given the different contexts for plasma testing (initial diagnosis *versus* progression), clear information on the request form and in the report are of utmost importance.

Several providers of external quality assessment (EQA) are already assessing reports as part of the proficiency testing program, based on the guideline by van Krieken et al. [11]. Results from these programs and a study in which routine reports were analyzed, already show the high variability between report content and layout [12]. To gain insight in the causes of this variability and how it can be improved this study was initiated as part of a national quality improvement project. There was a twofold aim: 1) to guide Belgian clinicians in providing the laboratory with sufficient information for biomarker testing and result interpretation and 2) to guide Belgian laboratories in reporting the results of biomarker analysis in a clinician-friendly way. Both aims apply to tissue testing and liquid biopsy testing for NSCLC.

## Methods

This study was subdivided in two major components; a) listing the ‘as is’ situation in selected Belgian laboratories, and b) analysis of opinions regarding requesting and reporting from both Belgian pathologists/molecular biologists and clinicians using a similar questionnaire.

1. *The total testing process in the visited laboratories*
  Four Belgian molecular pathology laboratories were included in the study based on their ISO 15189:2012 accreditation status for NSCLC testing on both tissue and plasma and their willingness to participate. Communication flows (from requesting to result reporting) were determined based on interviews with delegates from the molecular pathology laboratory.
2. *Defining what is currently included in requests and reports vs what should be according to guidelines*
  During a laboratory visit, the presence of pre-defined elements was studied on request form templates (*N* = 4) and reports (*N* = 4) using a checklist (see Additional file 1). Three documents were identified as applicable to both requesting molecular pathology tests and reporting test results in Belgium: a) a Royal Decree [13, 14], b) the code of practice for pathological anatomy [15] and c) ISO 15189:2012 [16]. In addition, European and international guidelines applicable to requesting and/or reporting were identified: a) a checklist developed by the College of American Pathologists [17] and b) a French national guideline [14]. These European and international guidelines are not legally applicable for Belgian laboratories, but since they have a stronger focus on non-small cell lung cancer they can be of educational value.
  For reporting only, data were also added from 2 EQA schemes for tissue testing (the Lung EQA scheme from the European Society of Pathology (ESP) of 2019 and the French national Gen&Tiss scheme of 2018–2019). In the molecular subscheme of the ESP Lung EQA scheme of 2019, laboratories submitted 1 molecular pathology report based on mock clinical information for a pre-selected case in which the *EGFR* c.2573 T > G; p.(Leu858Arg) and c.2369C > T; p.(Thr790Met) variants were present, as described elsewhere [18]. Similarly, the participants to the Gen&Tiss scheme of 2018–2019 submitted, in addition to their genotyping results, 1 molecular pathology report for a sample which harbored an *EGFR* exon 19 deletion [19].
3. *Relation between clinical interpretations and request form*
  The participating laboratories were asked to select nine reports and their corresponding request forms for tissue and eight for plasma testing based on pre-defined selection criteria. It was checked on all reports whether the clinical question on the request form was answered and whether this was done in a general or patient-specific way. General is hereby defined as ‘the effect of the presence/absence of a variant on therapy response, as observed during previous clinical trials’ and patient-specific by ‘an interpretation that only applies to the specific patient’.
4. *Content of request forms and reports—opinions from providers and receivers*

Two systems were used to study the opinions from the laboratory side (pathologists and molecular biologists) and of the clinician (pulmonologists, medical oncologists and radiotherapists): real-life voting during meetings/symposia and an online questionnaire. The real-life voting rounds were held as part of scientific symposia regarding NSCLC. Questions were presented during different sessions of national expert meetings between October 2018 and March 2019. The attendees had to answer the presented questions via an online voting system (PollEverywhere.com, Poll Everywhere Inc., San Francisco, Unites States). During each session, a set of basic questions were asked, which were extended with additional questions according to the target audience. An overview of the questions asked is shown in Additional file 2. In addition to the real-life voting sessions, a link to an online questionnaire (FormDesk software version 4.0.14, Innovero Software Solutions B.V., Wassenaar, the Netherlands) was spread via the Belgian AstraZeneca Newsletter of September 2019 (see Additional file 3).

## Results

**Fig. 1:**
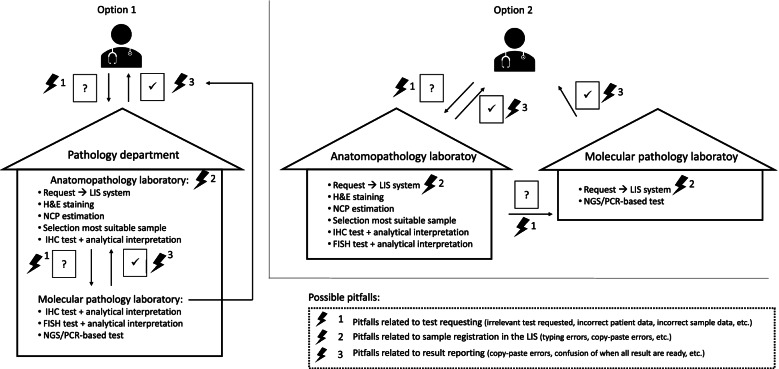
Communication flows and their pitfalls between pathologists/molecular biologists and clinician in Belgium. Legend: Option 1: the anatomic pathology- and molecular pathology laboratory are part of the same unit and located at the same place. Option 2: the anatomic pathology- and molecular pathology laboratory are distinct units and might have separate locations, LIS: laboratory information system, H&E: hematoxylin and eosin, NCP: neoplastic cell percentage, IHC: immunohistochemistry, FISH: fluorescent in-situ hybridization, NGS: next-generation sequencing, PCR: polymerase chain reaction

1. *The total testing process in the visited laboratories*
  In the visited hospitals, molecular pathology laboratories were a structural part of the 'pathology department' (*N* = 2) or part of a unit separate from the 'pathology department' (*N* = 2), whether or not as part of the center for human genetics (Fig. 1). In both structures, the entire testing process (requesting tests, performing tests and reporting test results) is different and there are other pitfalls. Table 1 shows the testing characteristics of the visited laboratories. In the majority, reflex testing and parallel testing co-existed, by choice of the laboratory or clinician. All laboratories tested for the *EFGR* gene in-house, but other genes could be outsourced. Most laboratories had a turnaround time between 8 and 14 calendar days.

**Table 1:** Characteristics of the study population

Characteristic	*N*	% (*N* = 4)
Type of testing		
Reflex testing	2	50%
Parallel testing	0	0%
Combination	1	25%
Clinician can choose	2	50%
Genes/proteins tested for NSCLC		
* EGFR*	4	100%
* ALK*	4	100%
* ROS1*	4	100%
* PD-L1*	4	100%
* KRAS*	4	100%
* BRAF*	4	100%
* MET*	4	100%
Average turnaround time (in calendar days)		
1 – 7	0	0%
8 – 14	3	75%
15 – 21	1	25%
> 21	0	0%
Tissue testing: in routine & accredited	4	100%
Liquid biopsy testing: in routine & accredited	4	100%
Input data from request form to the LIS		
Manually	3	75%
Automatically (via barcoding)	1	25%
2. *What is currently included in requests and reports vs what should be according to guidelines?*
  The presence of pre-defined elements was checked on four request forms and four reports for both tissue- and liquid biopsy testing during the laboratory visits (Tables 2 and 3).

**Table 2:** Presence of scored elements in request forms from visited Belgian laboratories versus elements required by guidelines/standards

Element	Present for tissue testing	Present for liquid biopsy testing	Required by Belgian standards [13,15,16,20]	Required by international guidelines [41]
**Request forms**	***N* = 4**	***N* = 4**		
***Accessibility and design***				
Molecular pathology dedicated form	**4 (100%)**	**1 (25%)**		
Lung cancer dedicated form	0 (0%)	0 (0%)		
Request available online	4 (100%)	4 (100%)		
Testing technique specified	4 (100%)	3 (75%)		
Online: summary of test methodology	2 (50%)	1 (25%)		
Online: indications for testing	2 (50%)	3 (75%)	✓	
Online: recipient	4 (100%)	4 (100%)	✓	
Online: transport medium	4 (100%)	NA	✓	
Online: max delay for transport	NA	4 (100%)	✓	
Online: storage	4 (100%)	4 (100%)	✓	
Digital storage completed forms	3 (75%)	3 (75%)		
***Administrative elements***				
Prescriber: name	4 (100%)	4 (100%)	✓	✓
Prescriber: address	2 (50%)	2 (50%)	✓	✓
Prescriber: RIZIV number or equivalent	4 (100%)	4 (100%)	✓	✓
Prescriber: department	1 (25%)	1 (25%)		✓
Reimbursement info	1 (25%)	2 (50%)		
General lab address	4 (100%)	4 (100%)		✓
Contact person in lab	1 (25%)	2 (50%)		
***Patient characteristics***				
Patient: name	4 (100%)	4 (100%)	✓	✓
Patient: DOB	4 (100%)	4 (100%)	✓	✓
Patient: gender	4 (100%)	4 (100%)	✓	✓
Patient: address	4 (100%)	4 (100%)	✓	✓
Patient: internal hospital ref	4 (100%	4 (100%)	✓	✓
Relevant patient history	**4 (100%)**	**2 (50%)**	✓	✓
Histology of tumor	2 (50%)	2 (50%)	✓	✓
Primary diagnosis	**1 (25%)**	**3 (75%)**		✓
Previous tests performed	3 (75%)	3 (75%)		✓
Original activating mutation	1 (25%)	2 (50%)		✓
Previous therapies (type)	**4 (0%)**	**1 (25%)**		✓
Previous therapies (time)	0 (0%)	0 (0%)		
Progression/not	3 (75%)	3 (75%)		✓
Progression type	1 (25%)	2 (50%)		
Progression time	0 (0%)	0 (0%)		
Location primary tumor	3 (75%)	3 (75%)		
Tumor stage	3 (75%)	3 (75%)		✓
Reason for testing	0 (0%)	1 (25%)	✓	✓
***Sample characteristics***				
Sample type	4 (100%)	4 (100%)	✓	✓
Number of slides/blocks/tubes	3 (75%)	4 (100%)		
Fixative	4 (100%)	NA		
Fixation time	4 (100%)	NA		
Specification of total blood/plasma	NA	0 (0%)		
Type of collection tube	NA	3 (75%)		
Date of sample collection	NA	3 (75%)	✓	
Time of sample collection	NA	3 (75%)	✓	

*Numbers in bold: elements for which a large difference (*≥ *30%) exists between tissue and liquid biopsy testing*

**Table 3:** Presence of scored elements in analyzed reports versus elements required by guidelines/standards

Element	Present for tissue testing			Present for liquid biopsy testing	Required by Belgian standards (13, 15, 16, 20)	Required by international guidelines (14, 17, 41, 42)
**REPORTS**	**Visited labs *N *= 4**	**ESP labs *N* = 86**	**G&T labs *N* = 47**	**Visited labs *N* = 4**		
***Administrative elements***						
Requesting physician: name	4 (100%)	77 (90%)	47 (100%)	4 (100%)	✓	✓
Requesting physician: address	4 (100%)	67 (78%)	**42 (89%)**	4 (100%)	✓	✓
Patient: name	4 (100%)	79 (92%)	47 (100%)	4 (100%)	✓	✓
Patient: address	2 (50%)	10 (12%)	1 (2%)	3 (75%)		
Patient: date of birth	4 (100%)	78 (91%)	46 (98%)	4 (100%)	✓	✓
Patient: gender	4 (100%)	71 (83%)	40 (85%)	4 (100%)		✓
Name report authorizer	4 (100%)	76 (88%)	40 (85%)	4 (100%)	✓	✓
Signature report authorizer	3 (75%)	63 (73%)	41 (87%)	3 (75%)		✓
Request date	2 (50%)	**48 (56%)**	32 (68%)	3 (75%)		✓
Sample collection date	4 (100%)	**61 (71%)**	46 (98%)	3 (75%)	✓	✓
Sample arrival date	3 (75%)	56 (65%)	45 (96%)	3 (75%)	✓	✓
Report validation date	4 (100%)	74 (86%)	42 (89%)	3 (75%)	✓	✓
Page nr/total pages	2 (50%)	55 (64%)	43 (91%)	2 (50%)	✓	✓
Concise titles of the analysis	2 (50%)	55 (64%)	45 (96%)	2 (50%)		✓
***Clinical information***						
Patient history	4 (100%)	76 (88%)	44 (94%)	4 (100%)	✓	✓
Planned line of therapy	**0 (0%)**	3 (3%)	6 (13%)	**2 (50%)**		✓
Reason for testing	2 (50%)	68 (79%)	21 (45%)	3 (75%)	✓	✓
***Sample characteristics***						
Sample type	4 (100%)	80 (93%)	38 (81%)	4 (100%)	✓	✓
Sample number	3 (75%)	83 (97%)	46 (98%)	3 (75%)		✓
% neoplastic cells	4 (100%)	77 (90%)	46 (98%)	NA		✓
ml blood analyzed	NA	NA	NA	1 (25%)		
***Method description***						
IVD/LDT	2 (50%)	16 (19%)	6 (13%)	1 (25%)		✓
DNA extraction method	**1 (25%)**	35 (41%)	26 (55%)	**3 (75%)**	✓	
Variant analysis method	4 (100%)	80 (93%)	47 (100%)	4 (100%)	✓	✓
Pre-analytical conditions	**2 (50%)**	**8 (9%)**	**2 (4%)**	**0 (0%)**	✓	✓
Sensitivity testing method	4 (100%)	69 (80%)	44 (94%)	4 (100%)		✓
Overview alterations tested	2 (50%)	79 (92%)	43 (91%)	3 (75%)	✓	✓
Reference sequence	2 (50%)	**44 (51%)**	7 (15%)	1 (25%)	✓	✓
***Results***						
Mutation status	4 (100%)	86 (100%)	47 (100%)	4 (100%)	✓	✓
Concentration extracted DNA	0 (0%)	8 (9%)	9 (19%)	1 (25%)		
VAF	1 (25%)	26 (30%)	15 (32%)	NA		
Disclaimer result validity	**1 (25%)**	1 (1%)	1 (2%)	**3 (75%)**		

* Numbers in bold: elements for which a large difference (*≥ *30%) exists between tissue and liquid biopsy testing. ESP: European Society of Pathology, G&T: Gen&Tiss (French national external quality assessment scheme)*
  Related to requesting, 19 of the scored elements are currently mandatory according to laws/standards applicable in Belgium [13, 20]. Of these required elements, only twelve for tissue and eleven for liquid biopsies were included by all four visited laboratories (marked in green in Table 2). Especially the availability of the indications for testing (for tissue), the address of the test prescriber, relevant patient history (for plasma), the tumor histology and the reason for testing are points of attention in the online laboratory guide (marked in red in Table 2).
  Similarly, eighteen elements are mandatory according to laws/standards valid in Belgium for reporting [13, 15, 20]. Of these, eleven and nine elements were included by all visited laboratories for tissue and liquid biopsy, respectively (marked in green in Table 3). Elements lacking most often on the report are the reason for testing (for tissue), the DNA extraction method, pre-analytical conditions, an overview of the alterations tested, the reference sequence and the total number of pages (marked in red in Table 3).
  Molecular pathology reports were submitted by 86 laboratories from 24 different countries as part of the molecular subscheme of the ESP Lung EQA scheme of 2019 and 47 laboratories during the Gen&Tiss scheme. Elements included in these reports from European laboratories are similar to the ones in the visited Belgian laboratories, except for some administrative elements (patient address, the date of sample arrival, the page numbering and the report title), the planned line of therapy, an overview of the alterations tested and the inclusion of the correct reference sequence of the tested genes (Table 3).
3. *Relation between interpretations and request form*
  The relation between the clinical question and the test result interpretation was checked on reports and their corresponding request forms for nine pre-defined situations for tissue testing and six for liquid biopsy testing (Table 4). It should be noted that not all laboratories had requests and reports for all pre-defined situations. For the majority of the testing situations, the clinical question was correctly answered on the report. However, for samples with *ALK* and *ROS1* rearrangements, this was often not the case because of an unclear question on the request form. For instance, in two hospitals the clinician often wrote only ‘*EGFR*?’ as a clinical question. It was observed that not for all cases a clear clinical question was present on the request form.

**Table 4:** Summary of opinions from pathologists/molecular biologists versus clinicians regarding requesting and reporting in Belgium

Question	Answers from the laboratory side	Answers from clinicians
**Elements on the request form**	**Which elements do you often miss? *N* = 18**	**Which elements do you always enter? *N* = 28**
Primary diagnosis	3 (17%)	28 (100%)
Previous tests performed	3 (17%)	12 (43%)
Original activating mutation	7 (39%)	19(68%)
Previous therapies	9 (50%)	11 (39%)
Tumor stage	4 (22%)	12 (43%)
Type of progression	4 (22%)	7 (25%)
Time of progression	3 (17%)	0 (0%)
Clinical question (reason for testing)	9 (50%)	21 (75%)
**In an ideal world: who is allowed to request additional tests?**	*N*= 21	*N*= 24
Only pathologist	2 (10%)	2 (8%)
Pathologist and clinician	18 (86%)	20 (83%)
Only clinician	1 (5%)	2 (8%)
Other	0 (0%)	0 (0%)
**Current situation: who is allowed to request additional tests?**	*N*= 18	*N*= 24
Only pathologist	1 (6%)	4 (17%)
Pathologist and clinician	9 (50%)	15 (63%)
Only clinician	7 (39%)	5 (21%)
Other (depends on the initial requester)	1 (6%)	0 (0%)
**Which of the following items regarding the request form are important?**	*N*= 0	*N*= 6
The request form is dedicated for molecular testing	-	4 (67%)
The request form is dedicated for specific cancer types	-	5 (83%)
An online version of the request form is available	-	0 (0%)
The indications for testing are indicated on the request form	-	6 (100%)
The testing techniques are indicated on the request form	-	6 (100%)
The tissue/blood recipient is indicated on the request form	-	5 (83%)
The transport medium is indicated on the request form	-	4 (67%)
The max delay for transport is mentioned on the request form	-	4 (67%)
**What is the reporting flow in your hospital?**	*N*= 18	*N*= 7
1 integrated report: biomarker results are added when available—WITH a conclusion per biomarker—WITH a conclusion on ALL biomarkers at the end	6 (33%)	3 (43%)
1 integrated report: biomarker results are added when available—WITH a conclusion per biomarker—WITHOUT a conclusion on ALL biomarkers at the end	8 (44%)	4 (57%)
1 integrated report: biomarker results are added when available—WITHOUT a conclusion per biomarker—WITH a conclusion on ALL biomarkers at the end	0 (0%)	0 (0%)
Separate reports are released for each biomarker result	3 (17%)	0 (0%)
Other	1 (6%)	0 (0%)
**How do you receive the report?**	*N*= 0	*N*= 7
On paper	-	3 (43%)
Via email	-	1 (14%)
Via the hospital information system	-	6 (86%)
Via telephone	-	0 (0%)
Other	-	0 (0%)
Question	Answers from the laboratory side	Answers from clinicians
**Elements on the result report**	**What do you think the clinician needs on the report? *N* = 19**	**What do you read on the report? *N *= 28**
Clinical interpretation	14 (74%)	27 (96%)
Description of the analytical method	1 (5%)	13 (46%)
Sensitivity of the test method	8 (42%)	13 (46%)
% neoplastic cells	4 (21%)	27 (96%)
Genotyping result	3 (16%)	18 (64%)
Tested regions of the target gene (e.g. which exons)	3 (16%)	15 (54%)
Variant allelic frequency	0 (0%)	1 (4%)
**Is it in your hospital appreciated by the clinician to give an interpretation on the test report?**	***N *= 4**	***N* = 4**
Yes, as a general interpretation (e.g. In general, patients with the L858R mutation in EGFR are sensitive to 1st and 2nd generation anti-EGFR TKI.)	3 (75%)	1 (25%)
Yes, as a direct advice (e.g. This patient has a L858R mutation in EGFR and should be treated with a 1st generation anti-EGFR TKI)	0 (0%)	2 (50%)
Yes, both as a general interpretation or direct advice	0 (0%)	1 (25%)
No, the clinician wants to make the interpretation	0 (0%)	0 (0%)
No, but for difficult cases the report is discussed with the clinician	0 (0%)	0 (0%)
I don't know	1 (25%)	0 (0%)
**Are some biomarker tests outsourced by your hospital?**	***N* = 6**	***N *= 3**
Yes	5 (83%)	3 (100%)
No	0 (0%)	0 (0%)
I don’t know	1 (17%)	0 (0%)
**What is the TAT for biomarker testing for NSCLC in your hospital?**	***N* = 6**	***N* = 3**
1–7 days	2 (33%)	3 (100%)
8–14 days	4 (67%)	0 (0%)
15–21 days	0 (0%)	0 (0%)
**What is the TAT for biomarker testing for NSCLC when tests are outsourced?**	***N* = 5**	***N* = 3**
1–7 days	0 (0%)	0 (0%)
8–14 days	5 (100%)	3 (100%)
15–21 days	0 (0%)	0 (0%)
4. *Content of request forms and reports—opinions from providers and receivers*

During several polling sessions the opinions of both the laboratory side (pathologists and molecular biologists) and the clinician were surveyed. The polling results from both groups are shown in Table 4. Elements that pathologists/molecular biologists were often missing on the request form (despite the fact that clinicians think they include it) were the original actionable variant (if applicable), previous therapies (if applicable) and a clear clinical question. Moreover, clinicians indicated that they wish to see more testing-related information on the request form, such as the indications for testing, the testing techniques and the recipients needed for sampling. For reporting, on the other hand, an item both considered important by both groups is the clinical interpretation of the test result. In addition, clinicians indicated that they also read the percentage of neoplastic cells.

## Discussion

For molecular pathology testing in the field of oncology, continuous and dynamic communication is fundamental between the laboratory and the clinician. When requesting the test in the pre-analytical phase, the laboratory expects the clinician to request the correct test and to provide sufficient information about the patient and the sample that was provided. In the post-analytical phase, it is important to formulate a correct answer to the clinician's question on the basis of the test results.

1. *The total testing process in the visited laboratories*
  Because of its different process steps (macroscopic evaluation, microscopic evaluation, DNA extraction, DNA testing, data processing and result interpretation), molecular pathology typically requires a more complex set-up than, for example, classical clinical chemistry. The communication flows differ between the visited laboratories, with anatomical- and molecular pathology being part of one department or being a physically separate department. Potential problems were identified in both contexts. Each time a test is requested, errors can be made by both the clinician (e.g. requesting the wrong test, writing incorrect patient information on the request form) [21, 22], the person registering the request into the laboratory information system (LIS) and by the pathologist requesting molecular testing in case the molecular pathology laboratory is physically separate (either by location or by LIS system). Some copy-paste or transfer errors might be prevented by providing digital request forms via the LIS [23]. It could be useful to attach the original pathology report to the request form for molecular testing, since this pathology rapport is likely to contain relevant patient information. Murphy et al. also identified several barriers for reporting the results to the clinicians, including the lack of a reliable process to contact clinicians [23] and to notify clinicians when all results are complete.
  Testing strategies differed among visited laboratories (Table 5). Although reflex testing was previously recommended [24], this is only done by two visited laboratories. Laboratories who used a combination of reflex and parallel testing often do their next-generation sequencing (NGS) testing in parallel with ALK and ROS1 IHC to reduce their turnaround time. In reflex to a positive ROS1 IHC result, a confirmatory FISH test is done, as this is still required according to the latest guidelines from the American Society of Clinical Oncology [25] and the European Society for Medical Oncology [26]. Aside from one laboratory, all have tested for *EGFR*, ALK, ROS1 and PD-L1, which are recommended according to current guidelines for NSCLC [25, 26]. These guidelines also concluded that multiplex platforms (NGS) are preferred over applying multiple targeted tests, due to the higher cost-effectiveness and the ability to detect more rare driver mutations which would stay under the radar by the classic reflex testing [25–29].

**Table 5:** Relationship between clinical question on the request form and the test result interpretation on the test report in Belgium

	Total	Type of interpretation			Reason for testing reflected in interpretation			Different lines of therapy considered			Discussion in tumor board advised		
		General	Direct	None	Yes	No	NA	Yes	No	NA	Yes	No	NA
Tissue testing													
Wild-type for ALK, ROS1 and *EGFR*	7	3 (43%)	1 (14%)	3 (43%)	3 (43%)	3 (14%)	0 (0%)	0 (0%)	4 (29%)	2 (71%)	0 (0%)	6 (100%)	0 (0%)
ALK positive	6	3 (50%)	1 (17%)	2 (33%)	2 (33%)	3 (67%)	0 (0%)	1 (17%)	5 (83%)	0 (0%)	0 (0%)	6 (100%)	0 (0%)
ROS1 positive	6	3 (50%)	1 (17%)	2 (33%)	2 (33%)	3 (67%)	0 (0%)	1 (17%)	4 (67%)	1 (17%)	0 (0%)	6 (100%)	0 (0%)
PD-L1 positive	5	4 (80%)	0 (0%)	1 (20%)	3 (60%)	2 (40%)	0 (0%)	2 (40%)	2 (40%)	1 (20%)	0 (0%)	5 (100%)	0 (0%)
* EGFR* exon 19del or 20/21 mutation at initial diagnosis	7	5 (71%)	1 (14%)	1 (14%)	4 (57%)	2 (29%)	0 (0%)	4 (57%)	3 (43%)	0 (0%)	0 (0%)	7 (100%)	0 (0%)
* EGFR* activating exon 19 deletion or exon 20 or 21 mutation positive with generalized disease progression	4	4 (100%)	0 (0%)	0 (0%)	3 (75%)	1 (25%)	0 (0%)	4 (100%)	0 (0%)	0 (0%)	0 (0%)	4 (100%)	0 (0%)
* EGFR* activating exon 19 deletion or exon 20 or 21 mutation and resistance mutation at disease progression	7	5 (71%)	1 (14%)	1 (14%)	4 (57%)	2 (29%)	0 (0%)	6 (86%)	1 (14%)	0 (0%)	0 (0%)	7 (100%)	0 (0%)
* EGFR* resistance mutation at disease progression (without initial activating mutation)	4	3 (75%)	1 (25%)	0 (0%)	4 (100%)	0 (0%)	0 (0%)	4 (100%)	0 (0%)	0 (0%)	0 (0%)	4 (100%)	0 (0%)
MET exon 14 skipping	2	2 (100%)	0 (0%)	0 (0%)	2 (100%)	0 (0%)	0 (0%)	0 (0%)	1 (50%)	1 (50%)	0 (0%)	2 (100%)	0 (0%)
Liquid biopsy testing													
* EGFR* WT	4	3 (75%)	1 (25%)	0 (0%)	3 (75%)	1 (25%)	0 (0%)	2 (50%)	0 (0%)	2 (50%)	0 (0%)	4 (100%)	0 (0%)
* EGFR* exon 19del or 20/21 mutation at initial diagnosis	3	1 (33%)	2 (67%)	0 (0%)	1 (33%)	2 (67%)	0 (0%)	3 (100%)	0 (0%)	0 (0%)	0 (0%)	3 (100%)	0 (0%)
* EGFR* exon 19 deletion or exon 20 or 21 mutation and resistance mutation at disease progression	4	2 (50%)	2 (50%)	0 (0%)	3 (75%)	1 (25%)	0 (0%)	4 (100%)	0 (0%)	0 (0%)	0 (0%)	4 (100%)	0 (0%)
* EGFR* resistance mutation at disease progression (without initial mutation)	2	1 (50%)	1 (50%)	0 (0%)	2 (100%)	0 (0%)	0 (0%)	2 (100%)	0 (0%)	0 (0%)	0 (0%)	2 (100%)	0 (0%)
* EGFR* exon 19 deletion or exon 20 or 21 mutation positive result after disease progression	3	2 (67	1 (67%)	0 (0%)	3 (100%)	0 (0%)	0 (0%)	3 (100%)	0 (0%)	0 (0%)	0 (0%)	3 (100%)	0 (0%)
Borderline T790M	1	1 (100	0 (0%)	0 (0%)	1 (100%)	0 (0%)	0 (0%)	1 (100%)	0 (0%)	0 (0%)	0 (0%)	1 (100%)	0 (0%)
2. *What is currently included vs what should be according to guidelines?*
  Although (inter)national guidelines and standards for test requesting and reporting exist, these are often interpretable in different ways and not transferrable to molecular pathology. Although ISO 15189:2012 is an international standard, it was categorized as a standard ‘applicable in Belgium’ in Tables 1 and 2 since Belgian laboratories have to be accredited for most molecular tests according to a national reimbursement law [13]. Overall, the analyzed forms and reports comply well with the guidelines applicable in Belgium (Tables 2 and 3). Required elements that were often missing on the request forms include the address and the national reimbursement (RIZIV) number of the prescriber, patient gender and address and the histology of the tumor. The tumor histology is not literally required by ISO15189:2012, but can be an interpretation of ‘the anatomic site of origin’ [16]. For reporting, sample arrival dates, page numbering, the DNA extraction method, pre-analytical conditions, overview of the variants tested and reference sequence were included by less than 70% of the laboratories.
  At first sight, the analyzed template request forms for tissue (N = 4) do not closely resemble the template forms for liquid biopsies (N = 4). Major differences between the seven requests analyzed for tissue testing and the five for liquid biopsy testing included: whether the form is dedicated for molecular pathology or not, the name of the test prescriber, the contact person in the laboratory, the primary diagnosis of the patient, the original actionable variant and whether there is progression or not. The latter was included in 80% of the laboratories, which could be expected since plasma testing for *EGFR* variants is often done in the context of resistance to 1^st^ and 2^nd^ generation anti-EGFR TKIs. Nevertheless, when resistance mutations, such as the p.(Thr790Met) variant, could not be detected [26], the test should be repeated on a tissue sample. Since these resistance mutations can be present at a low variant allelic frequency [30], knowing that the patient progressed might impact the testing strategy and could thus be a clinically relevant item on the request form. Regardless of the differences between tissue and liquid biopsies, there are also items that are missing in both. A first example is the exact reason for testing. It would seem logical that the molecular laboratory can only give a conclusion when a question is asked, but in the majority of cases this is not explicitly formulated. Secondly, the progression type—and time of progression are also often lacking. As mentioned earlier, this is especially relevant for liquid biopsy testing. At the end of the molecular pathology report or during a molecular tumor board meeting, a conclusion has to be made in terms of therapy; which may differ between full-, local- and oligo progression [26].
  In addition to the analysis of the request forms, opinions from both the laboratory and requester side were gathered (Table 4). Although the number of participants was too low, a discrepancy seems to exist between what clinicians claimed they always enter on the request form versus what pathologists often miss, especially for the primary diagnosis, previous test performed, original actionable mutations and clinical question.
  It was also analyzed whether sufficient information regarding the testing process was available online. In most laboratories, this was already the case for the sample recipient and transport medium, but not for the test specifications and indications for testing (Table 2). In addition, the surveys show that the majority of clinicians would also like to see the indications, techniques, containers, transport media and transport delay on the application form (Table 4).
  For the analyzed reports, the differences between tissue and liquid biopsy testing are less prominent. More abundant in the liquid biopsy reports are the request date, the planned line of therapy, the DNA extraction method, pre-analytical conditions, the reference sequence and a note regarding the result validity. This is to be expected somehow, since the previously mentioned testing context for progression and also because the pre-analytical conditions have more impact on the test result for liquid biopsies [31–33]. Of the elements that are present in both report types (page numbering, concise title, planned line of therapy and whether the method is IVD/LDT), only the page numbering is crucial for the report, since loss of a page with critical information of the test can lead to wrong treatment decisions [4].
  Overall, the elements described in the reports from the visited Belgian laboratories did not differ substantially from the reports submitted during EQA (Table 3). However, these four laboratories had all participated in the ESP EQA scheme at least once, which might bias this observation. Nevertheless, several discrepancies existed. The patient’s address is less frequently included in EQA. There could be 2 possible explanations; 1) this information is in routine often retrieved from the online patient record, which is not available for EQA or 2) this data may have been anonymized for EQA purposes. Elements included at a higher frequency in the French reports are the presence of a title, the page numbering and the sample arrival date. This could be because French laboratories lose points if these elements are missed during the Gen&Tiss EQA scheme. A similar effect is seen for the overview of alterations tested and the reference sequence for the ESP EQA scheme.
  In a similar way as for the request forms, we surveyed laboratory- and clinicians’ opinions regarding reporting. Both groups agree that the clinical interpretation is a critical element on the report. It might surprise pathologists, but clinicians also want to know the percentage of neoplastic cells, although this is a rather technical aspect. It must be noted that the genotyping result is not read by all, which emphasizes that the clinical interpretation must be certainly correct.
  Based on these study results, proposed templates for requesting and reporting are shown in Additional file 3 and 3.
3. *Relation between interpretations and request form*
  One of the aims of the study was to determine whether the question asked on the request form was fully answered in the report in 4 Belgium laboratories. Reports were studied for various clinical situations that are representative for NSCLC testing for tissue and liquid biopsies (Table 5) [26, 34]. It was checked whether a clinical interpretation was given and whether this was written in a ‘general’ or ‘direct’ way. By general is meant, for example: “in general, patients with an activating mutation in the *EGFR* gene show response to 1^st^ and 2^nd^ generation anti-EGFR TKIs”, whereas ‘direct’ can be rather “this patient will respond to gefitinib”. The majority of laboratories gave a general interpretation. No guidelines regarding general interpretations exist, but this is recommended by several external quality assessment providers such as the European Society of Pathology. For ALK-, ROS1- and PD-L1 positive results, the reason for testing was not always reflected. For certain cases the request form only stated “*EGFR*?”. The laboratories claimed that they know the oncologist meant “test all relevant biomarkers for NSCLC”, among which ALK, ROS1 and PD-L1. For *EGFR* plasma testing at initial diagnosis, it was unclear in two laboratories whether progression occurred and therefore both interpretations for first- and second-line therapy were given. Especially in the interpretations for *EGFR*-positive cases (at initial diagnosis and after progression) different lines of therapy were considered. This makes sense in view of treatment with osimertinib which can be prescribed when the patient has a resistant mutation such as the p.(Thr790Met) variant [26]. Note that at the time the study was conducted, osimertinib was not yet approved for first-line treatment. Whether the test result should be discussed in a molecular tumor board is not mentioned on any report. In Belgium, the implementation of molecular tumor boards is still under development. This may be because multidisciplinary oncological consults per cancer type are already mandatory for each patient before therapy can be reimbursed and the need for an additional molecular tumor board, during which only rare gene variants are discussed, therefore seems less important till now [13, 35]. However, several recent studies indicated that MTBs specifically facilitate the interpretation of the molecular test results and a treatment advice on this result regarding rare, uncommon and complex molecular results, and MTBs have improved further the patient outcome [35, 36]. In 2020, the International Society of Liquid Biopsy recommended the implementation of a MTB in the clinical workflow of liquid biopsy testing to interpret NGS results [37].
4. *Content of request forms and reports—opinions from providers and receivers*

Apart from a clear content of the request forms and reports, clear "peripheral communication" is important for a smooth process. Given the limited number of molecular pathology laboratories, the group of persons attending the meetings during which their opinions were polled was rather limited. Nevertheless, some trends could be observed. For example, the majority would like both pathologists and clinicians to request tests, which is not always the case now (Table 4). This indicates that there could be a perception that pathologists are more aware of the relevance of certain tests in certain situations. Previous studies in genetics indicated indeed that clinicians do not always feel confident about their choice of test [38, 39]. In most laboratories clinicians receive the report via the hospital information system. A possible hazard is that in the majority of cases reports are built in this system as integrated reports, but without a single conclusion at the end. It is then the responsibility of the clinician to make a therapy decision based on all single test results, which can be challenging. Based on literature, reporting in a timely manner was found to be burdensome [40], but the survey results indicated that clinicians rather under- than overestimate the turnaround time (Table 4).

## Conclusions

In conclusion, this study identified different communication flows between the laboratory (both anatomical- and molecular pathology) and the clinician, different content in request forms and result reports and different opinions from both pathologists and clinicians in Belgium. Although the number of visited laboratories and polled pathologists and clinicians was rather limited, several trends were clear which add to the need for standardization of the test requesting and reporting process in Belgium. Existing guidelines and standards on the content of test request forms and result reports are vague, yet not all elements were included in the visited laboratories. The interpretation is the most important element of the report and should contain an answer to the question asked in the request. Based on the study results in Belgium, a template for complete requesting and reporting was proposed, which could harmonize communication flows. The reports analyzed during EQA showed similar results. Nevertheless, it should be further studied to which extent EQA reports resemble routine reports. In addition, automatization of the flow might reduce typing/copy-pasting errors. Therefore, the authors also wish to communicate these observations to programmers/vendors of electronic forms/reporting system so the necessary elements can be implemented.

## Supplementary Information

**Additional file 1: Supplementary file 1**

**Additional file 2: Supplementary file 2**

**Additional file 3: Supplementary file 3**

**Additional file 4: Supplementary file 4**

## Data Availability

The datasets used and/or analyzed during the current study are available from the corresponding author on reasonable request.

## Abbreviations

ALK: Anaplastic lymphatic kinase
BRAF: B-Raf murine sarcoma viral oncogene homolog B
ddPCR: Digital droplet polymerase chain reaction
DOB: Date of birth
EGFR: Epidermal growth factor receptor
EQA: External quality assessment
ESP: European Society of Pathology
FISH: Fluorescence in situ hybridisation
G&T: Gen&Tiss
H&E: Hematoxylin and eosin
IHC: Immunohistochemistry
ISO: International organisation for standardisation
IVD: In vitro diagnostic
KRAS: Kirsten rat sarcoma viral oncogene homolog
LB: Liquid Biopsy
LDT: Laboratory developed test
LIS: Laboratory information system
MET: Mesenchymal to Epithelial Transition
NCP: Neoplastic cell percentage
NGS: Next generation sequencing
NSCLC: Non-small-cell lung cancer
PCR: Polymerase chain reaction
PDL1: Programmed deadth ligand 1
QC: Quality control
RECIST: Response evaluation criteria in solid tumors
ROS1: ROS Proto-Oncogene 1
TKI: Tyrosin kinase inhibitor
TNM: Tumor, Nodes and Metastases
VAF: Variant allelic frequency
WT: Wild type
