# Supplementary material for: Molecular pathology testing for non-small cell lung cancer: an observational study of elements currently present in request forms and result reports and the opinion of different stakeholders
Source: BMC Cancer. 2022 Jul 6;22:736. doi: 10.1186/s12885-022-09798-5 (PMC9258204; doi:10.1186/s12885-022-09798-5)
Supplement: Supplementary file 1 — Additional file 1: Supplementary file 1 [file 12885_2022_9798_MOESM1_ESM.docx]

***Additional file1 – checklist used to score the presence of elements on request forms and reports in Belgian laboratories***

REQUEST FORMS

1. Accessibility and design

| Tissue | | Liquid biopsy | |
| --- | --- | --- | --- |
| Molecular pathology dedicated form? | ☐ Yes ☐No | Liquid biopsy dedicated form? | ☐ Yes ☐No |
| Lung cancer dedicated form? | ☐ Yes ☐No | Lung cancer dedicated form? | ☐ Yes ☐No |
| Online version available? | ☐ Yes ☐No | Online version available? | ☐ Yes ☐No |
| Testing technique specified on form? | ☐ Yes ☐No | Testing technique specified on form? | ☐ Yes ☐No |
| Link to test specifications on form? | ☐ Yes ☐No | Link to test specifications on form? | ☐ Yes ☐No |
| Test specifications online available? |  | Test specifications online available? |  |
| Indications for testing | ☐ Yes ☐No | Indications for testing | ☐ Yes ☐No |
| Recipient | ☐ Yes ☐No | Type of tubes | ☐ Yes ☐No |
| Transport medium | ☐ Yes ☐No | Max delay for transport | ☐ Yes ☐No |
| Storage | ☐ Yes ☐No | Storage | ☐ Yes ☐No |
| Digital storage of completed forms? | ☐ Yes ☐No | Digital storage of completed forms? | ☐ Yes ☐No |

1. Administrative elements

| Item | Tissue | Liquid biopsy |
| --- | --- | --- |
| Prescriber details |  |  |
| Name | ☐ Yes ☐No | ☐ Yes ☐No |
| Address | ☐ Yes ☐No | ☐ Yes ☐No |
| RIZIV-number | ☐ Yes ☐No | ☐ Yes ☐No |
| Department | ☐ Yes ☐No | ☐ Yes ☐No |
| Reimbursement info | ☐ Yes ☐No | ☐ Yes ☐No |
| General lab address | ☐ Yes ☐No | ☐ Yes ☐No |
| Contact person | ☐ Yes ☐No | ☐ Yes ☐No |

1. Patient characteristics

| Item | Tissue | Liquid biopsy |
| --- | --- | --- |
| Patient identification |  |  |
| Name | ☐ Yes ☐No | ☐ Yes ☐No |
| Date of birth | ☐ Yes ☐No | ☐ Yes ☐No |
| Gender | ☐ Yes ☐No | ☐ Yes ☐No |
| Address | ☐ Yes ☐No | ☐ Yes ☐No |
| Internal hospital reference | ☐ Yes ☐No | ☐ Yes ☐No |
| Clinical information |  |  |
| Relevant patient history | ☐ Yes ☐No | ☐ Yes ☐No |
| Histology of tumor | ☐ Yes ☐No | ☐ Yes ☐No |
| Primary diagnosis | ☐ Yes ☐No | ☐ Yes ☐No |
| Previous tests performed | ☐ Yes ☐No | ☐ Yes ☐No |
| Original activating mutation | ☐ Yes ☐No | ☐ Yes ☐No |
| Previous therapies |  |  |
| Type of anti-*EGFR* TKI | ☐ Yes ☐No | ☐ Yes ☐No |
| Time after anti-*EGFR* TKI | ☐ Yes ☐No | ☐ Yes ☐No |
| Progression |  |  |
| Progression or not | ☐ Yes ☐No | ☐ Yes ☐No |
| Type of progression | ☐ Yes ☐No | ☐ Yes ☐No |
| Time of progression | ☐ Yes ☐No | ☐ Yes ☐No |
| Location of primary tumor | ☐ Yes ☐No | ☐ Yes ☐No |
| Tumor stage |  |  |
| Grade I,II,III,IV | ☐ Yes ☐No | ☐ Yes ☐No |
| TNM stage | ☐ Yes ☐No | ☐ Yes ☐No |
| Primary or metastatic | ☐ Yes ☐No | ☐ Yes ☐No |
| RECIST data | ☐ Yes ☐No | ☐ Yes ☐No |
| Reason for testing | ☐ Yes ☐No | ☐ Yes ☐No |
| Sample characteristics |  |  |
| Sample type | ☐ Yes ☐No | ☐ Yes ☐No |
| Number of slides/blocks/tubes | ☐ Yes ☐No | ☐ Yes ☐No |
| Tissue: fixative | ☐ Yes ☐No |  |
| Tissue: time of fixation | ☐ Yes ☐No |  |
| LB: specification total blood/plasma |  | ☐ Yes ☐No |
| LB: Type of collection tube |  | ☐ Yes ☐No |
| LB: date of sample collection |  | ☐ Yes ☐No |
| LB: time of sample collection |  | ☐ Yes ☐No |

REPORTS

1. Administrative elements

| Item | Tissue | Liquid biopsy |
| --- | --- | --- |
| Requesting physician |  |  |
| Name | ☐ Yes ☐No | ☐ Yes ☐No |
| Address | ☐ Yes ☐No | ☐ Yes ☐No |
| Patient |  |  |
| Name | ☐ Yes ☐No | ☐ Yes ☐No |
| Address | ☐ Yes ☐No | ☐ Yes ☐No |
| Date of birth | ☐ Yes ☐No | ☐ Yes ☐No |
| Gender | ☐ Yes ☐No | ☐ Yes ☐No |
| Report authorizer |  |  |
| Name | ☐ Yes ☐No | ☐ Yes ☐No |
| Signature | ☐ Yes ☐No | ☐ Yes ☐No |
| Dates |  |  |
| Request date | ☐ Yes ☐No | ☐ Yes ☐No |
| Sample collection | ☐ Yes ☐No | ☐ Yes ☐No |
| Sample arrival | ☐ Yes ☐No | ☐ Yes ☐No |
| Report authorization | ☐ Yes ☐No | ☐ Yes ☐No |
| Page number/total number of pages | ☐ Yes ☐No | ☐ Yes ☐No |
| Concise title of the analysis | ☐ Yes ☐No | ☐ Yes ☐No |

1. Clinical information

| Item | Tissue | Liquid biopsy |
| --- | --- | --- |
| Patient history |  |  |
| Copied from the request form | ☐ Yes ☐No | ☐ Yes ☐No |
| Summarized | ☐ Yes ☐No | ☐ Yes ☐No |
| Planned line of therapy | ☐ Yes ☐No | ☐ Yes ☐No |
| Reason for testing | ☐ Yes ☐No | ☐ Yes ☐No |

1. Sample characteristics

| Item | Tissue | Liquid biopsy |
| --- | --- | --- |
| Sample type | ☐ Yes ☐No | ☐ Yes ☐No |
| Sample number | ☐ Yes ☐No | ☐ Yes ☐No |
| Tissue: % neoplastic cells | ☐ Yes ☐No |  |
| LB: Milliliter blood analyzed |  | ☐ Yes ☐No |

1. Method description

| Item | Tissue | Liquid biopsy |
| --- | --- | --- |
| Method clearly mentioned (kit and supplier) |  |  |
| IVD or LDT mentioned | ☐ Yes ☐No | ☐ Yes ☐No |
| DNA extraction method | ☐ Yes ☐No | ☐ Yes ☐No |
| Mutation analysis method | ☐ Yes ☐No | ☐ Yes ☐No |
| Pre-analytical conditions | ☐ Yes ☐No | ☐ Yes ☐No |
| Sensitivity of the test method (+ NGS QC metrics) | ☐ Yes ☐No | ☐ Yes ☐No |
| Overview of mutations analyzed |  |  |
| Activating/resistance mutations | ☐ Yes ☐No | ☐ Yes ☐No |
| Exons only | ☐ Yes ☐No | ☐ Yes ☐No |
| Codons only | ☐ Yes ☐No | ☐ Yes ☐No |
| Specific variants | ☐ Yes ☐No | ☐ Yes ☐No |
| Reference sequence | ☐ Yes ☐No | ☐ Yes ☐No |

1. Results

| Item | Tissue | Liquid biopsy |
| --- | --- | --- |
| Mutation status clearly mentioned | ☐ Yes ☐No | ☐ Yes ☐No |
| Concentration of extracted DNA | ☐ Yes ☐No | ☐ Yes ☐No |
| Tissue: VAF | ☐ Yes ☐No |  |
| LB: disclaimer ‘a WT does not exclude the presence of a mutation’ |  | ☐ Yes ☐No |
| Tissue: disclaimer ‘result validity’ | ☐ Yes ☐No |  |
