# Supplementary material for: Molecular pathology testing for non-small cell lung cancer: an observational study of elements currently present in request forms and result reports and the opinion of different stakeholders
Source: BMC Cancer. 2022 Jul 6;22:736. doi: 10.1186/s12885-022-09798-5 (PMC9258204; doi:10.1186/s12885-022-09798-5)
Supplement: Supplementary file 2 — Additional file 2: Supplementary file 2 [file 12885_2022_9798_MOESM2_ESM.docx]

**Additional file 2 – overview of the Belgian polling sessions and the questions asked during these sessions**

| Questions | Pathologists | | | | Clinicians | | |
| --- | --- | --- | --- | --- | --- | --- | --- |
|  | Session 1 | Session 2 | Session 3 | Session 4 | | Session 5 | Session 6 |
| What is the reporting flow in your hospital? | x | x | x |  | | x | x |
| Which clinical information do you often miss on the request form? | x | x | x |  | |  |  |
| Which clinical information do you always enter on the request form? |  |  |  | x | | x | x |
| Which info on the request form do you think is essential for the laboratory to perform the correct test and give a correct interpretation? |  |  |  |  | | x |  |
| Which elements on the report do you think clinicians need for correctly interpreting the test result? | x | x | x |  | |  |  |
| Which elements on the report are included in your institution? |  |  | x |  | |  |  |
| What do you always read on the report? |  |  |  | x | | x | x |
| What do you never read on the report? |  |  |  |  | | x | x |
| In an ideal world: who is allowed to request additional tests? | x | x | x | x | | x | x |
| Current situation: who is allowed to request additional tests? | x | x | x | x | | x | x |
| Are some biomarker tests outsourced by your hospital? |  | x |  |  | | x |  |
| What is the TAT for biomarker testing for NSCLC in your hospital? |  | x |  | x | | x |  |
| What is the TAT for biomarker testing for NSCLC when you outsource tests? |  | x |  |  | | x |  |
| How important are the following items regarding the request form? |  |  |  |  | | x | x |
| How do you receive the report? |  |  |  |  | | x | x |
| Is it in your hospital appreciated by the clinician to give an interpretation on the test report? |  |  | x |  | | x | x |

***Session 1:*** “Solving the puzzle of diagnostic testing in NSCLC patients”, Belgian Week of Pathology, Brussels, Belgium. ***Session 2:*** Forpath workshop “Working with Big Medical DATA in Daily Medical Practice”, Brussels, Belgium. ***Session 3:*** online questionnaire distributed to pathologists via the AstraZeneca newsletter. ***Session 4:*** Satellite symposium “Maximizing the potential of new treatment paradigms in NSCLC”, La Hulpe, Belgium. ***Session 5:*** 7th multidisciplinary meeting thoracic oncology, Oostkamp, Belgium. ***Session 6:*** online questionnaire distributed to clinicians via the AstraZeneca newsletter
