# Supplementary material for: Molecular pathology testing for non-small cell lung cancer: an observational study of elements currently present in request forms and result reports and the opinion of different stakeholders
Source: BMC Cancer. 2022 Jul 6;22:736. doi: 10.1186/s12885-022-09798-5 (PMC9258204; doi:10.1186/s12885-022-09798-5)
Supplement: Supplementary file 3 — Additional file 3: Supplementary file 3 [file 12885_2022_9798_MOESM3_ESM.docx]

Laboratory name
Street name
City

Telephone number, Email

Date of request:
../../….

Laboratory
logo

**ADMINISTRATIVE INFORMATION**

Name requester: …
RIZIV number requester: …

Tel number requester: …
Signature requester: …

Patient name: …
Patient DOB: …
Patient gender : …

Patient hospital ID : …

**TEST INDICATION**

Localization primary tumor: Stage: ☐ primary ☐ metastasis
Clinical context: ☐ diagnostic Previously tested? ☐ No
 ☐ monitoring ☐ Yes: ………….
 ☐ progression: ☐ local EGFR-TKI given? ☐ No
 ☐ oligo ☐ Yes: ………….

Comments: ………………………………………………………………………………………………………………………………………

**SAMPLE INFORMATION**

Sample number:
Sample type:
☐ tumor tissue Tumor histology: …
 Collection date: ../../…. Collection time: ../../….
 Fixative: ☐ 4% buffered formaldehyde ☐ other: …
 Fixation time: ☐ 4 – 24h ☐ 24 – 48h ☐ > 48h

☐ plasma Collection date: ../../…. Collection time: ../../….
 Number of tubes …

**ST INDICATION**

**REQUESTED TESTS**

| ☐ | Reflex testing:   - Mutation analysis (NGS): ALK, BRAF, CDKN2A, DDR2, EGFR, ERBB2, KRAS, MET (exon 14 skipping), PIK3CA, RET and ROS1 - ALK IHC, if +: FISH confirmation - ROS1 IHC, if +: FISH confirmation - PD-L1 IHC | ☐ | ALK IHC, if +: FISH confirmation |
| --- | --- | --- | --- |
|  |  | ☐ | ROS1 IHC, if +: FISH confirmation |
|  |  | ☐ | PD-L1 IHC |
|  |  | ☐ | c-MET gene amplification (FISH) |
| ☐ | Mutation analysis (NGS): ALK, BRAF, CDKN2A, DDR2, EGFR, ERBB2, KRAS, MET (exon 14 skipping), PIK3CA, RET and ROS1 | ☐ | RET translocation (FISH) |
|  |  | ☐ | *EGFR* mutation analysis with ddPCR in plasma |

Further instructions:

*For tissue samples:*
 Send biopsy after collection fixation in phosphate buffered formaldehyde 4% on
 room temperature and forward to the molecular pathology laboratory together with the request.
*For plasma samples:*
 Collect blood with Streck tubes and fill tubes completely. Immediately mix by
 gently inverting the tube 10 times. Store and transport the tubes at room temperature.
