# Supplementary material for: Molecular pathology testing for non-small cell lung cancer: an observational study of elements currently present in request forms and result reports and the opinion of different stakeholders
Source: BMC Cancer. 2022 Jul 6;22:736. doi: 10.1186/s12885-022-09798-5 (PMC9258204; doi:10.1186/s12885-022-09798-5)
Supplement: Supplementary file 4 — Additional file 4: Supplementary file 4 [file 12885_2022_9798_MOESM4_ESM.docx]

Authorized report
Version:

Report date: ../../….

Laboratory name
Street name
City

Telephone number
Email

Laboratory
logo

Page 1/1

**ADMINISTRATIVE INFORMATION**

Name requester :
Address requester :

Patient name:
Patient DOB:
Patient gender :

Patient hospital ID :

Sample ID (internal) : Sample collection date :
Sample ID (external) : Sample arrival date :

Sample type :

**TEST INDICATION**

**Diagnosis:** NSCLC stage IV

**Patient history:** Previous tumor biopsy: ☐
 Previous mutation:
 Previous therapy:
 Progression:

**Request for:** Mutation analysis for targeted therapies

**TEST METHOD**

**DNA extraction method:** Kit name (supplier)  **Test method:** Principle: e.g. NGS
 Panel name (supplier)
 Sequencer (supplier)

**Tested regions of the target gene:**
*EGFR* exon 19 deletions, *EGFR* c.2369C>T, p.(Thr790Met), *EGFR* c.1474A>C, p.(Ser492Arg) c.2155G>A, p.(Gly719Ser) c.2155G>T, p.(Gly719Cys) c.2156G>C, p.(Gly719Ala) c.2159C>T, p.(Ser720Phe) c.2303G>T, p.(Ser768Ile), c.2327G>A, p.(Arg776His) c.2369C>T, p.(Thr790Met) c.2390G>C, p.(Cys797Ser) c.2573T>G, p.(Leu858Arg) c.2573_2574TG>GT, p.(Leu858Arg) c.2582T>A, p.(Leu861Gln)
Reference sequence of *EGFR*: NM_005228.x

**Sensitivity of the method:** x% mutated DNA in a wild-type background

**TEST RESULT**

**Percentage neoplastic cells in sample:** x%

**Genotyping results:** mutation detected in [gene], c…., p.(…)

**INTERPRETATION TEST RESULT**

Example: The primary activating *EGFR* exon 19 del confers sensitivity to first- and second-generation anti-*EGFR* TKI, but the c.2369C>T, p.(Thr790Met) variant might cause resistance. However, patients with the c.2369C>T, p.(Thr790Met) variant might be sensitive to third generation anti-*EGFR* TKI.

Pathologist: Name Report authorizer: Name
 Signature Signature
